# Unusual clinical manifestations and predominant stopgain *ATM* gene variants in a single centre cohort of ataxia telangiectasia from North India

**DOI:** 10.1038/s41598-022-08019-0

**Published:** 2022-03-08

**Authors:** Amit Rawat, Rahul Tyagi, Himanshi Chaudhary, Vignesh Pandiarajan, Ankur Kumar Jindal, Deepti Suri, Anju Gupta, Madhubala Sharma, Kanika Arora, Amanjit Bal, Priyanka Madaan, Lokesh Saini, Jitendra Kumar Sahu, Yumi Ogura, Tamaki Kato, Kohsuke Imai, Shigeaki Nonoyama, Surjit Singh

**Affiliations:** 1 Allergy and Immunology Laboratory, Department of Pediatrics, Advanced Pediatric Centre, Postgraduate Institute of Medical Education and Research (PGIMER), Chandigarh, 160012 India; 2 Department of Histopathology, Postgraduate Institute of Medical Education and Research, Chandigarh, India; 3 Pediatric Neurology Unit, Department of Pediatrics, Post Graduate Institute of Medical Education and Research, Chandigarh, India; 4 National Defense Medical College (Japan), Saitama, Japan; 5 Tokyo Medical and Dental University, Tokyo, Japan

**Keywords:** Clinical genetics, Medical genetics, Sequencing, Immunogenetics, Immunological disorders, Genetics, Immunology, Neurology

## Abstract

Germline *ATM* gene variations result in phenotypic heterogeneity characterized by a variable degree of disease severity. We retrospectively collected clinical, genetic, and immunological data of 26 cases with A-T. Clinical manifestations included oculocutaneous telangiectasia (100%), ataxia (100%), fever, loose stools or infection (67%), cerebellar atrophy (50%), nystagmus (8%), dysarthria (15.38%), and visual impairment (8%). Genetic analysis confirmed *ATM* gene variations in 16 unrelated cases. The most common type of variation was stopgain variants (56%). Immunoglobulin profile indicated reduced IgA, IgG, and IgM in 94%, 50%, and 20% cases, respectively. T cell lymphopenia was observed in 80% of cases among those investigated. Unusual presentations included an EBV-associated smooth muscle tumour located in the liver in one case and Hyper IgM syndrome-like presentation in two cases. Increased immunosenescence was observed in T-cell subsets (CD4+CD57+ and CD8+CD57+). T-cell receptor excision circles (TRECs) were reduced in 3/8 (37.50%) cases.

## Introduction

Ataxia-telangiectasia (A-T) is an early-onset autosomal recessive inborn error of immunity (IEI) characterized by a constellation of symptoms including progressive debilitating neuro-degeneration, cerebellar ataxia, oculocutaneous telangiectasia, recurrent sino-pulmonary infections, radiation sensitivity, insulin-resistant diabetes, and a high risk of lymphoid malignancies. The worldwide prevalence of A-T has been estimated to vary from 1 in 40,000 to 1 in 100,000^1,2^. The disorder is caused by a loss of function variants in the ataxia telangiectasia mutated (*ATM*) gene, which was mapped to human chromosome 11 at 11q22. 3 loci with 66 exons (with 62 coding exons; NM_000051.4) spreading across 150 kb of genomic DNA^3,4^.

Mutations in the *ATM* gene lead to alteration/truncation of the encoded ATM protein, a Serine/Threonine (Ser/Thr) protein kinase, which belongs to the phosphatidyl inositol (PI) 3/4‐kinase family. Ser/Thr protein kinase acts as a DNA damage sensor protein in the nucleus and activates checkpoint signaling upon exposure to genotoxic stressors like ionizing radiation. Individuals with A-T are exquisitely sensitive to ionizing radiation that can lead to double-stranded breaks (DSBs) in DNA. The ATM protein is also involved in maintaining mitochondrial stability and glucose homeostasis in the cytoplasm. A wide range of clinical (clinical manifestations, laboratory features, imaging, and pathological) heterogeneity has been observed in patients with A-T, which suggests high genotypic heterogeneity or the role of epigenetic factors^5^ or both.

Leiden open variation database lists 5625 public variants in the *ATM* gene in different populations (last updated-February 022). Understanding the genetic variants and their effect on ATM protein may be helpful in a better understanding of the disease process and developing a patient-specific management strategy. In earlier studies from India, Malviya et al. described immunological abnormalities in five families with A-T in 1973^6^. Even though there are numerous reports on the clinical profile of A-T; there is a dearth of literature describing the spectrum of genetic abnormalities in patients with A-T from India.

Earlier studies have reported abnormal immunoglobulin levels with discernible IgA deficiency and reduced IgE and IgG. A-T with affected ATM Kinase functions has been reported to have severe immunological outcomes^7^. However, comparatively milder immunological alterations have been correlated as a result of the residual presence of ATM protein^7,8^. Generally, the presence of truncating variants resulted in severe forms of A-T due to the absence of ATM protein.

We report retrospective data revealing clinical, genetic, and immune profiles of the North Indian cohort with ataxia telangiectasia.

## Methodology

### Participants

Clinical data were collated from records of patients who were diagnosed as A-T in the Pediatric immunodeficiency clinic and/or Pediatric Neurodevelopmental clinic of the Advanced Pediatrics Centre, Postgraduate Institute of Medical Education and Research (PGIMER), Chandigarh, India. The patient files were retrieved and the data was recorded on a predesigned proforma. The study was approved by the department review board (DRB) of Advanced Pediatric Centre, PGIMER, Chandigarh, India (no. DRB-21-21).

### Clinical and laboratory investigations

Laboratory investigations performed included complete blood counts (CBCs), immunoglobulin levels (IgG, IgA, and IgM), IgG subclasses (IgG1, G2, G3, and G4), neuroimaging (Computed tomography or Magnetic resonance imaging of the brain), and serum alpha-fetoprotein levels. Lymphocyte and B cell subset analysis was performed in a few patients using flow cytometry. T cell (T-helper and T-cytotoxic) immunosenescence was also estimated in two recently examined cases. T-cell receptor excision circles (TRECs), Coding joint-kappa-deleting recombination excision circles (cjKREC) and signal joint KREC (sjKREC) levels were determined as reported previously^9^. RNase-P was used as an internal control. Epstein–Barr virus-associated smooth muscle tumour was examined by Epstein–Barr encoding region-in situ hybridization (EBER-ISH) in one case.

### Genetic investigations

Genomic DNA was isolated from EDTA blood samples using QIAmp DNA Blood Mini kit (Qiagen, Germany) as per the manufacturer’s instructions. Genetic investigations could be carried out in 16 unrelated cases. Genetic analysis in 5 A-T cases was performed at the National Defense Medical College, Japan using a targeted NGS gene panel containing 29 genes implicated in severe combined immunodeficiency and all coding exons of the *ATM* gene with ion semiconductor sequencing and multiplex PCR amplicons. In the remaining cases, next-generation sequencing was performed using a custom-made panel comprising 44 common PID genes including all coding exons of the *ATM* gene at the Allergy Immunology Laboratory, Department of Pediatrics, PGIMER, Chandigarh. Briefly, the targeted gene panel consisting of the *ATM* gene was designed using Ion AmpliSeq Designer (ThermoFisher Scientific, USA). Genomic DNA was quantified using QubitTM Fluorometer (ThermoFisher Scientific, USA) followed by target amplification using PID 2 × 2-primer pool panel. The amplified product was partially digested followed by adapter ligation, barcoding, library purification, and amplification. Further, DNA fragments were immobilized on an ion sphere particle and clonally amplified using Ion One TouchTM 2 instrument. The emulsion PCR results in beads containing amplified and cloned DNA fragments. Elimination of empty beads was done using a robotic enrichment system. Sequencing was performed using Ion S5 instrument and simultaneously processed on an Ion torrent server for assembly and further analysis. Variant calling and analysis of results were made using Ion reporter software (ThermoFisher Scientific, USA). As large deletions and duplications are not detected by this method, BAM files of the patients were also analysed on Integrative Genomics Viewer (IGV) software to check for large deletion/duplication; Supplementary Fig. 1. The identified variants were validated using Sanger sequencing. We have described the *ATM* variants based on transcript NM_000051.4. Novelty of variants was determined using Human Gene mutation database (HGMD), 1000 genomes database, Genome Aggregation Database (gnomAD) browser. The effect of pathogenic variants on the protein structure and functions was predicted using in-silico tools (Mutation Taster 2.0, PROVEAN, SIFT, and PolyPhen2). SPLICE AI, MaxEnt, and dbscSNV scores were obtained to predict the effect of splicing variants through ensembl variant effect predictor (https://www.ensembl.org/Tools/VEP). MoBiDiC Prioritization Algorithm (MPA) scores were also obtained to predict the splicing defect.

### Statistical analysis

Data were recorded on an MS Excel^®^ sheet and analyzed using the SPSS Statistic 21 (IBM SPSS Statistics, New York, USA). The results were expressed as percentages and as mean–standard deviation (SD) for quantitative variables.

### Ethics approval

The study protocols were approved and performed in accordance with the guidelines outlined by departmental review board of Advanced Pediatrics Centre, PGIMER, Chandigarh, India (Vide no. DRB-21-21).

### Consent to participate

Written informed consent was obtained from participants in the manuscript, wherever required. In case of minors the consent was obtained from the legally authorised representative.

### Consent for publication

Due consent taken for publication of clinical photographs and other clinical images. In case of minors the consent was obtained from the legally authorised representative.

## Results

### Clinical presentation

Twenty six patients were clinically diagnosed with A-T during the study period. Patient details have been presented in Table 1. Of these, 15 were females (57.70%) and 11 were males (42.30%). The mean age at onset of symptoms was 2 years (SD 0.88; range 9 months to 3 years). However, the mean age at diagnosis was 7 years (SD 3.01; range 2–12).

**Table 1:** Baseline characteristics of A-T cases.

Variables	Values
Total A-T cases (unrelated cases)	26 (25)
Age in years: mean (SD; range)	7 (3.01; 2–12)
Gender (M:F)	11:15
Weight (at presentation) in kg: mean (SD)	15 (6.3)
Height (in cm): mean (SD)	110 (14.56)
Alpha-fetoprotein levels in ng/ml (normal < 10): median (range)	178 (52.68–611.9)
Hb (in g/dL): mean (SD)	11 (2.33)
Platelet count (× 10^9^/L): mean (SD; range)	333 (218; 21.4–712)
Total leukocyte count (TLC) (× 10^9^/L): mean (SD; range)	8.5 (3.3; 4.1–13.35)
Absolute neutrophil count (ANC) (× 10^9^/L): mean (SD; range)	4.8 (2.8; 1.1–8.5)
Absolute lymphocyte count (ALC) (× 10^9^/L): mean (SD; range)	2.4 (1.2; 0.5–5.8)
Confirmed A-T cases with *ATM* gene variants	16
Confirmed A-T cases with homozygous variants: n (%)	9 (56.25%)
Confirmed A-T cases with compound heterozygous variants: n (%)	7 (43.75%)
Positive family history in confirmed cases: n (%)	6 (37.50%)
Consanguinity in confirmed cases: n (%)	5 (31.25%)
Total number of variants	14

**Figure 1:**
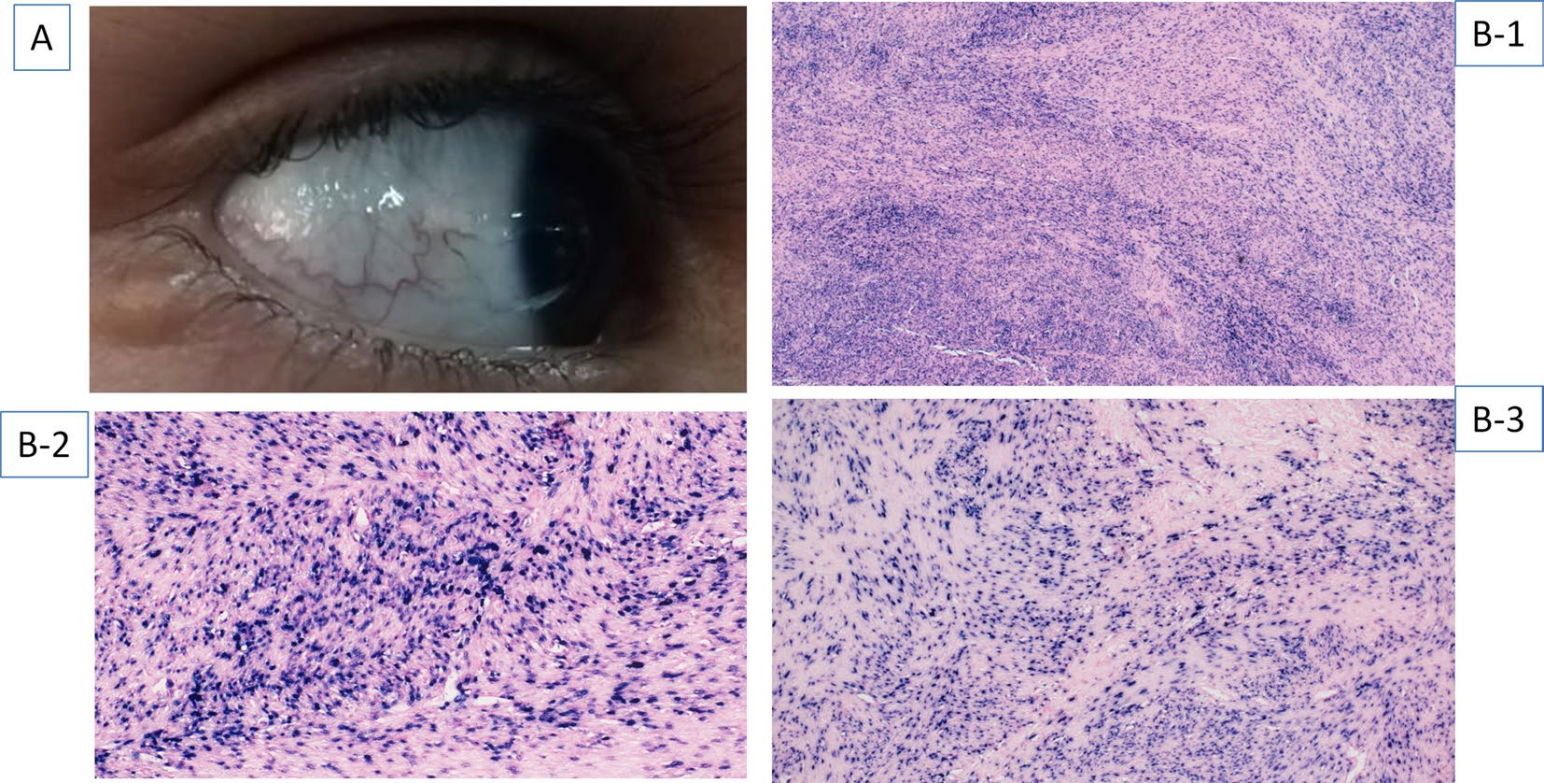
(**A**) Ocular Telangiectasia in the P-15 with Hyper IgM-like phenotype. (**B1**–**B3**) Analysis of EBV-induced smooth muscle tumor in P-16 by Epstein–Barr encoding region (EBER) in situ hybridization (ISH).

A family history of sibling death because of recurrent infections was noted in 3 patients. Parental consanguinity was reported in 5 patients. Two of the patients were related. Ocular or oculocutaneous telangiectasia was present in all patients; Fig. 1A. Some patients presented with unusual but previously reported clinical manifestations. One of the cases (P16) presented with a smooth muscle tumor (SMT) located in the liver, which was confirmed to be an EBV-associated SMT (by EBER ISH). This tumor was initially misdiagnosed as a leiomyoma on routine histopathological examination (Fig. 1B1–3). Median alpha-fetoprotein (AFP) level was 178.60 ng/ml (range 52.68–611.9 ng/ml). In addition, 2 patients (P8, P15) presented with recurrent infections and an immunological phenotype mimicking Hyper IgM syndrome. The detailed clinical profile has been provided in Table 2.

**Table 2:** Clinical details of 26 patients with A-T.

Case	1	2	3	4	5^#^	6^#^	7	8	9	10	11	12	13	14	15	16	17	18	19	20	21	22	23	24	25	26
Gender	M	F	M	M	F	M	F	F	F	F	F	F	F	F	F	M	F	M	M	F	M	M	F	F	M	M
Age (years)	5.5	12	9	3	3.5	2	6	10	3	7	9	4	8	8	6	2	9	9	8	7	12	7	3	10	8	11
Age of onset (years)	3	2	1.8	0.9	0.9	0.9	2	2.5	1.5	3	2	1.8	2	1.1	2	0.9	3	3	3	1.1	2.8	0.6	1.6	3.5	1.8	2
Fever/cough episode	+	−	−	−	−	−	+	−	−	−	−	−	−	−	+	+	+	+	+	+	+	+	+	+	+	+
Loose stools	+	−	−	−	−	−	+	−	−	−	−	−	−	−	−	+	−	−	−	−	+	−	−	−	−	−
Recurrent infections	−	+	−	−	−	+	−	−	−	−	−	−	−	−	+	+	+	+	+	+	+	+	+	+	+	+
Immunodeficiency	+	*	+	*	*	*	+	+	+	+	*	+	*	*	+	+	+	+	*	*	+	+	*	*	*	−
Gait abnormality	+	+	+	+	+	+	+	+	+	+	+	+	+	+	+	+	+	+	+	+	+	+	+	+	+	+
Swaying while sitting	−	−	−	+	+	+	−	−	−	−	−	−	−	−	−	−	−	−	−	−	−	+	+	−	−	+
Ocular telangiectasia	+	+	+	+	+	+	+	+	+	+	+	+	+	+	+	+	+	+	+	+	+	+	+	+	+	+
Pallor	+	−	−	+	+	+	+	−	−	−	−	−	−	−	−	−	+	−	−	−	−	−	−	−	−	+
Nystagmus	−	+	−	−	−	−	+	−	−	−	−	−	−	−	−	−	−	−	−	−	−	−	−	−	−	+
Cerebellar atrophy	+	+	−	−	*	+	−	−	−	+	+	+	−	*	*	−	+	+	−	−	+	+	−	*	*	+
Visual impairment	−	−	−	−	−	−	−	−	−	−	+	−	−	−	−	−	−	−	−	−	−	+^$^	−	−	−	−
Dysarthria	+	+	−	−	−	−	−	−	−	−	+	−	−	−	−	−	−	−	−	−	−	−	−	−	−	+

^#^Siblings (in adjacent columns). *Data not available. ^$^Retinitis pigmentosa.

### Immunodeficiency in A-T

A history suggestive of immune defect i.e., recurrent infections, fevers, and loose stool were noted in 14/26 (53.8%) patients. Lymphopenia was noted in all cases (100%) with a mean absolute lymphocyte count (ALC) of 2.4 × 10^9^/l (± 1.2 SD). Neutropenia was noted in only 10% of patients. Immunoglobulin profile indicated reduced IgA, IgG, and IgM in 88.23% (15/17), 46.66% (7/15), and 18.18% (2/11) cases, respectively. Elevated levels of IgM were observed in 4/11 (36.36%). Among the genetically confirmed cases, immunoglobulin A (IgA) and IgG levels were assessed in 11 patients. Among these, IgA and IgG were reduced in 10/11 (90.90%) and 5/11 (45.45%) patients, respectively. IgM levels were assessed only in 8 cases and levels were found to be reduced only in one case (14.28%) but elevated in 4/8 (50%). IgG subclasses were analyzed in five cases. IgG1 was found to be reduced (P7 and P8) in two cases and markedly enhanced in one case (P3). IgG2 was reduced in three cases (P1, P7 and P9). Two patients (P8, P15) presented with elevated IgM and decreased level of IgG and A, conforming to a phenotype of Hyper IgM syndrome (HIGM). One of these two patients also had severe T cell lymphopenia. Both these children had recurrent infections since early infancy. Both the patients were initially diagnosed and managed as Hyper IgM syndrome. They developed ataxia and ocular telangiectasia later in life, following which genetic testing confirmed the diagnosis of A-T. Retinitis pigmentosa was observed in one case who also had recurrent infections and respiratory tract infections.

**Figure 2:**
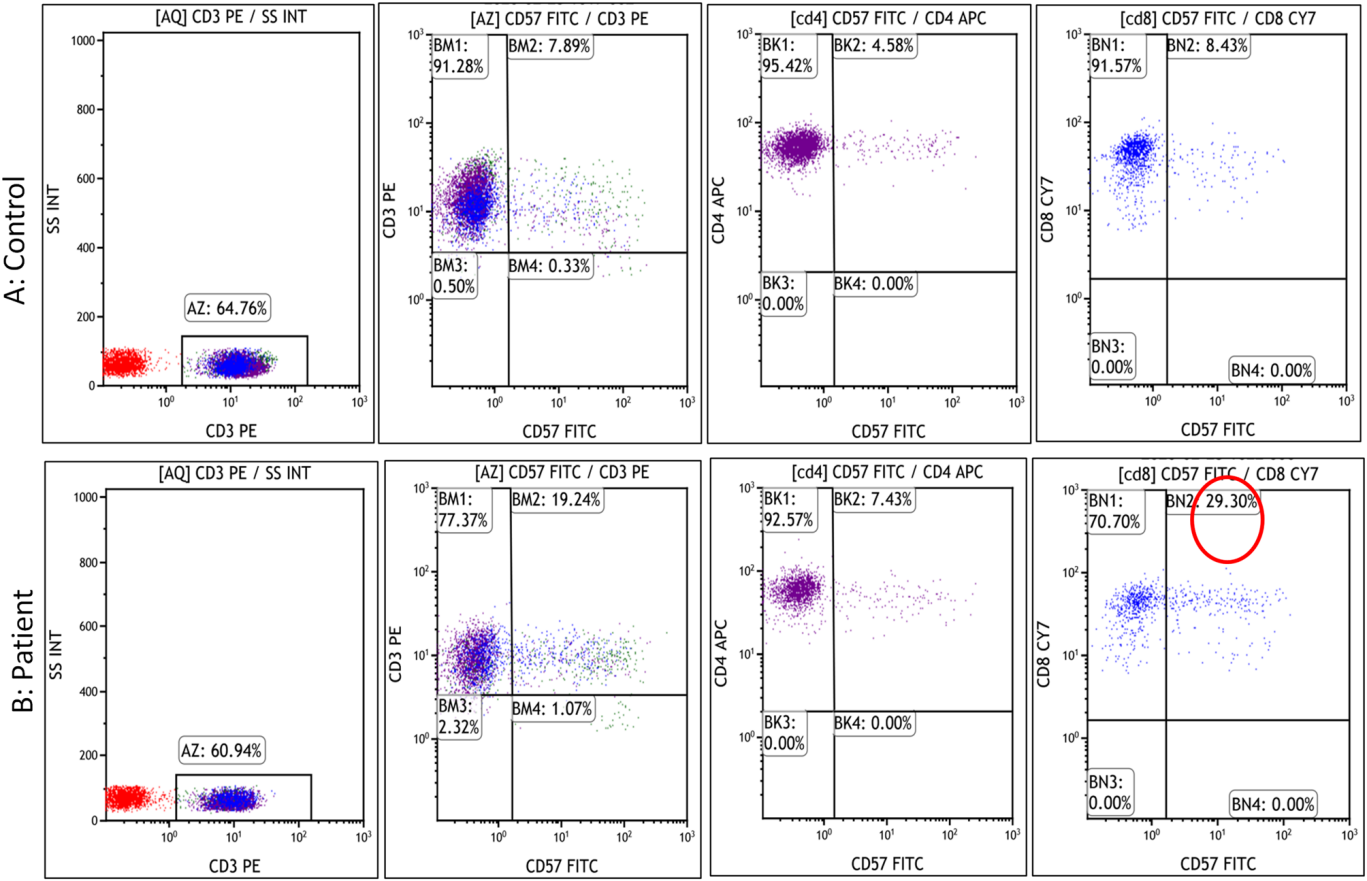
Representative image showing flow cytometric analysis of immunosenescent T lymphocytes in control (**A**) and patient (**B**). Elevated expression of CD57 marker revealed immunosenescence in the CD4+ (7.43% in comparison to the control 4.58%) and CD8+ T cells (29.30% in comparison to the control 8.43%). CD3+ T cells (constituting both CD4+ T helper and CD8+ T cytotoxic cells) were gated on CD3 vs SSc. Further, immunosenescence was estimated on the CD4+ and CD8+ cells.

Analysis of lymphocyte subsets in eight A-T cases revealed reduced proportions of CD3+ cells in 7/8 (87.50%) [P7: 55.67%; P9: 51.87%, P16:25.69%; P17:54.89%, P18: 56.72%; P22: 45% and P26: 58%). However, CD19+ ve B cells were found to be reduced in 3/8 cases (P8: 1.59%: P16: 2.06%, P26: 3.44%). The representative flow cytometry results have been supplied in Supplementary Fig. 2. B cell immunophenotyping was performed in two cases (P15, P18). In P15, switched B lymphocytes (CD19+IgM−IgD−) were strikingly reduced (2.72%) in comparison to the control (25.05%) indicating a severe switching defect Supplementary Fig. 3. The proportion of CD19+CD27+IgD− switched memory B cell was found to be 8.81% in comparison to the control 25.69%; Supplementary Fig. 3. A marked increase in immunosenescent T lymphocytes was also observed (CD4+CD57+ and CD8+CD57+) in two cases (P15 and P18) as shown in Fig. 2.

### TREC analysis

**Figure 3:**
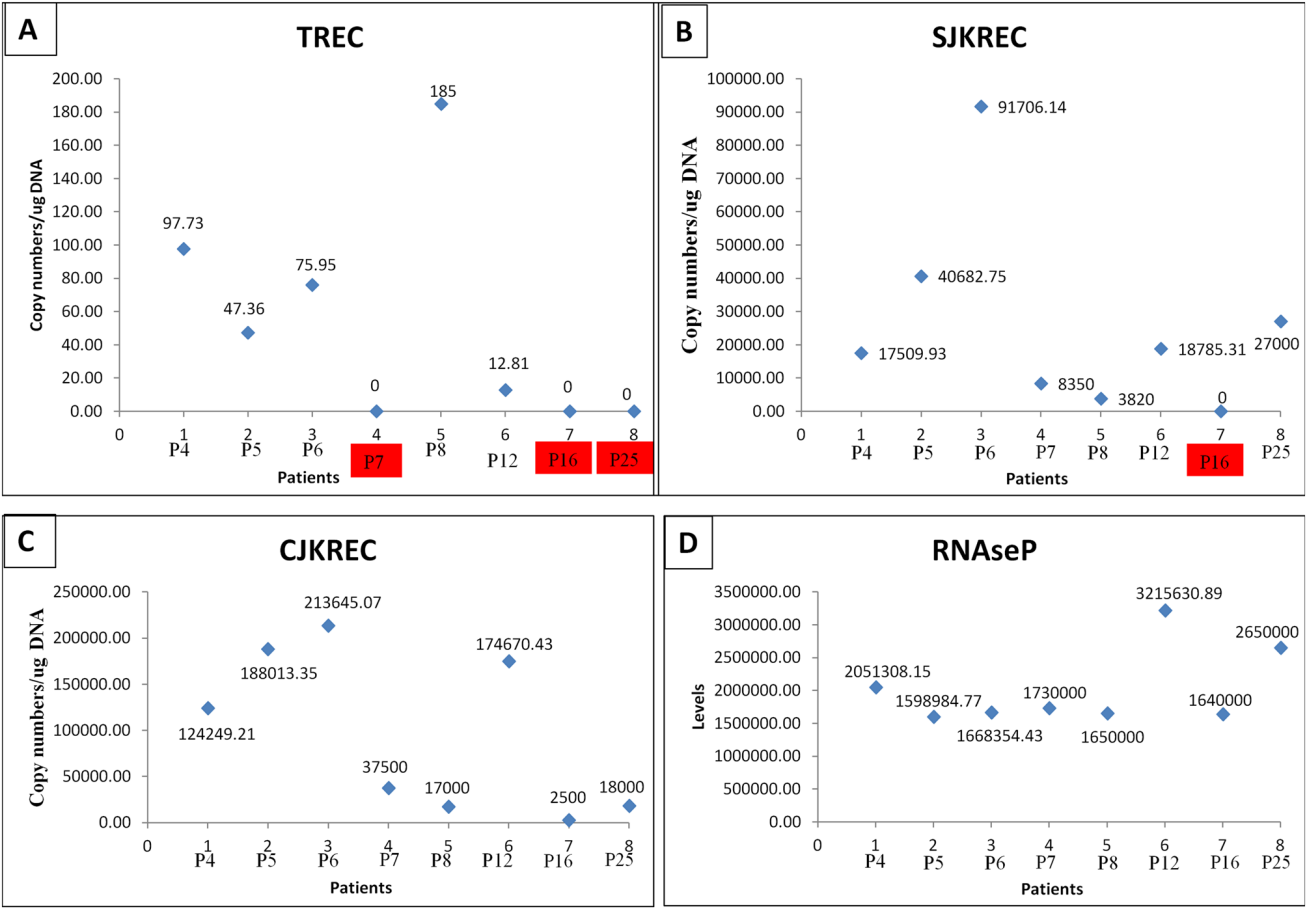
Scatter plot representing the copy number values of (**A**) TREC, (**B**) sjKRECs, (**C**) cjKRECs and (**D**) RNAseP as internal control. P represents number of case along with the values. X axis: Serial numbers of cases. Y axis: Copy numbers of TREC, sjKRECs, cjKREC and RNaseP per µg DNA.

TREC analysis could be conducted in eight AT cases. Among these TREC were negative in three cases (P7, 16 and P25). SJ-KRECs were also negative in case P16 indicating altered B-cell neogenesis. The scatter plot representing the TREC, sjKRECs and cjKRECs have been provided in the Fig. 3, Supplementary Table 1.

### Genetic analysis

**Figure 4:**
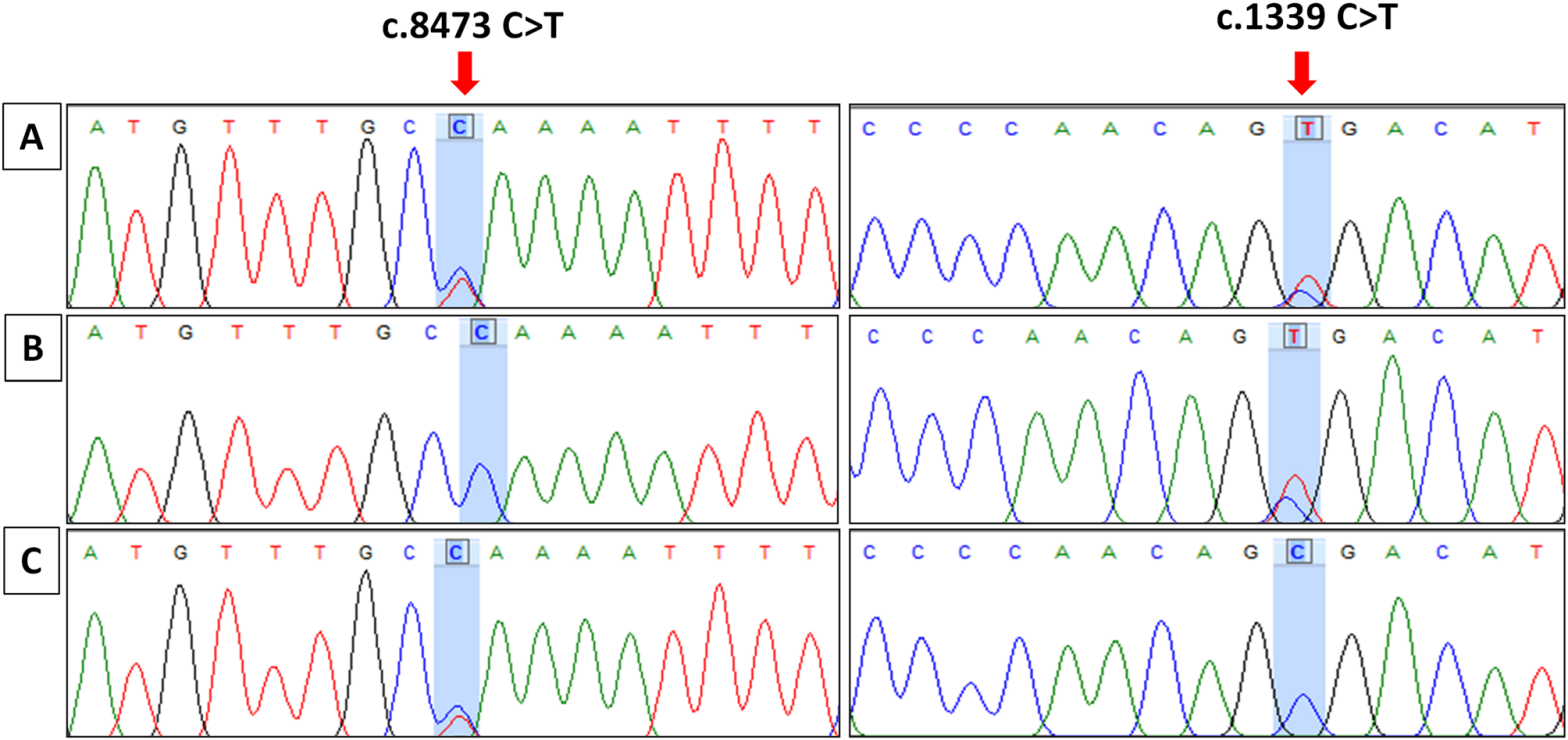
Representative electropherogram of compound heterozygote variants in *ATM* gene. (**A**) Chromatogram of Patient reveals heterozygous nonsense variant at c.8473C>T position resulting in termination of translation event at residue 2825, and c.1339 C>T variant in another allele leading to premature termination at residue 447. (**B**) Chromatogram of father revealed wild type genotype at c.8473 location (C/C) and heterozygous C/T variation at c.1339 position in another allele. (**C**) Chromatogram of mother revealed heterozygous C/T variant at c.8473 position and wild type C/C genotype at c.1339 position.

*ATM* gene variants were found in 16 out of 25 clinically diagnosed unrelated A-T cases (including a sibling pair). Among these 68.75% (11/16) had homozygous, whereas 31.25% (5/16) had compound heterozygous *ATM* gene variations. Representative chromatograms revealing compound heterozygous variants in P11 describing trans locations are provided in Fig. 4. In nine cases, genetic investigations could not be performed. These cases were clinically diagnosed when facilities for genetic diagnosis were not available. Karyotyping to detect 7, 14 translocations could not be performed in these patients and most of these patients were lost to follow-up. However, clinical features of patients without a genetic diagnosis were not different from those with a confirmed molecular diagnosis. All diagnosed patients either based on clinical criteria or with a confirmed molecular diagnosis had characteristic features of ataxia, ocular telangiectasia, and increased levels of alpha fetoprotein.

**Figure 5:**
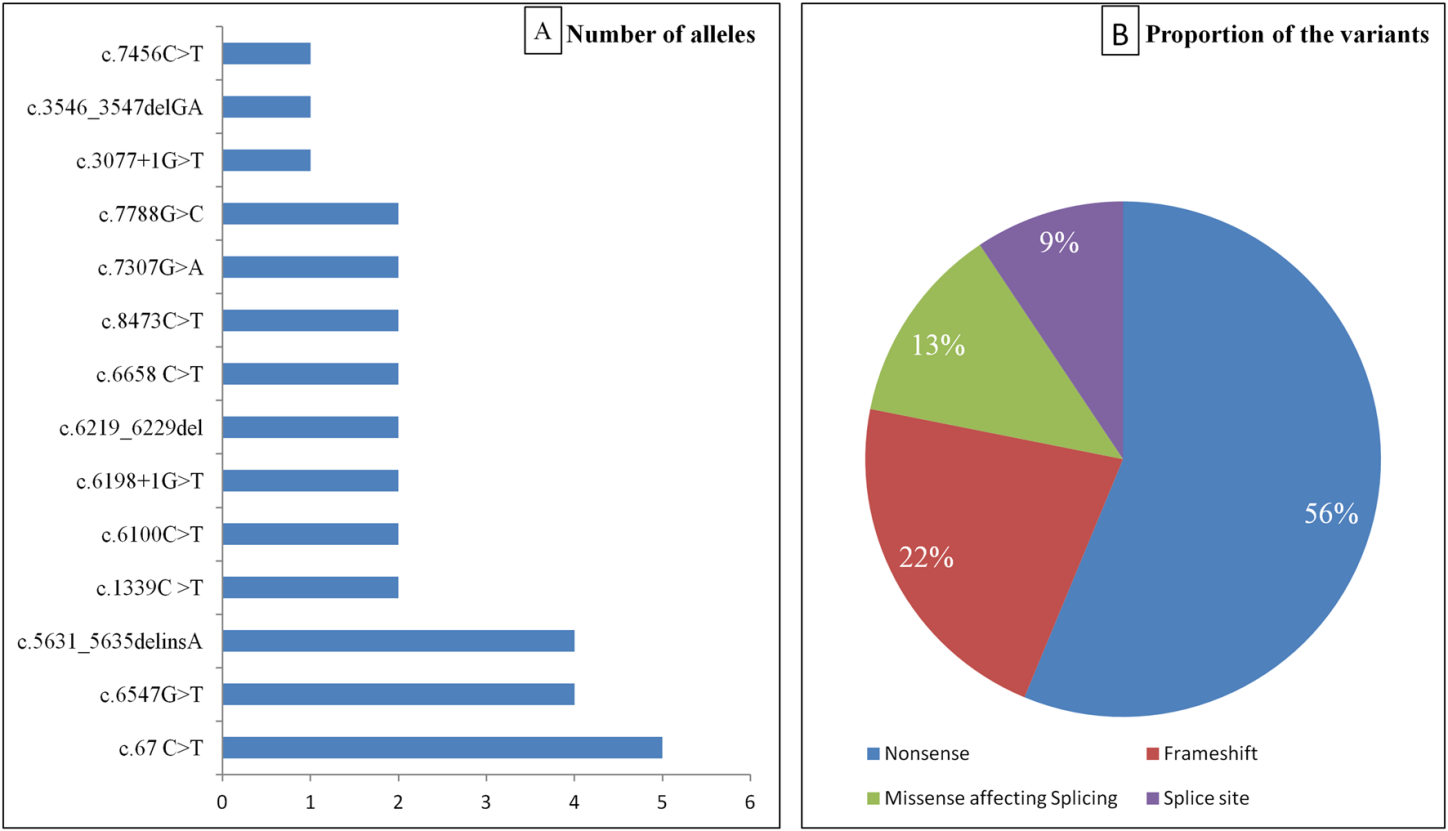
Frequency and effect of various mutations. (**A**) Number of alleles among 16 unrelated cases (32 alleles). (**B**) Proportion of variants according to the effect on the protein.

Among 16 unrelated cases (32 alleles) 14 different variants were found. The details of variants have been provided in the Table 3. The most frequent variant was c.67C>T [5/32 (15.62%)] followed by c.6547G>T [4/32 (12.50%)], and c.5631_5635delCTCGCinsA [4/32 (12.50%)]. The details of variant wise frequency have been provided in Fig. 5A. Premature termination (Stopgain) was found to be the most common genetic variation, present in 56% of cases followed by frameshift (22%), Missense (13%) and splicing variants (9%). Two missense variants were found that probably disrupt the splicing site according to the in silico tools provided in Supplementary Table 2. Unfortunately, these predictions have not been confirmed by published transcriptional studies. Figure 5B depicts the distribution of various types of variants among A-T cases.

**Table 3:** Spectrum of *ATM* gene variants in the North Indian patients with A-T.

Case no	Zygosity	Variants				Affected ATM protein domain	Clinical significance*
		Exon	cDNA nucleotide change	Protein change	Type		
3	CH	24	c.3546_3547delGA	p.Asn1183TrpfsTer16	Frameshift	HEAT	Pathogenic
		50	c.7456C>T	p.Arg2486Ter	Nonsense	FAT	Pathogenic
4	H	45	c.6547G>T	p.Glu2183Ter	Nonsense	FAT	Pathogenic
5^#^	H	45	c.6547 G>T	p.Glu2183Ter	Nonsense	FAT	Pathogenic
6^#^	H	45	c.6547 G>T	p.Glu2183Ter	Nonsense	FAT	Pathogenic
7	CH	10	c.1339C>T	p.Arg447Ter	Nonsense	HEAT	Pathogenic
		58	c.8473C>T	p.Gln2825Ter	Nonsense	PI3K	Pathogenic
8	H	Intron 42–43	c.6198+1G>T	Splice site variant	Donor splicing variant	FAT	Likely pathogenic
9	H	2	c.67 C>T	p. Arg23Ter	Nonsense	N-terminal-TAN	Pathogenic
10	H	46	c.6658 C>T	p.Gln2220Ter	Nonsense	FAT	Pathogenic
11	CH	10	c.1339C>T	p.Arg447Ter	Nonsense	HEAT	Pathogenic
		58	c.8473C>T	p.Gln2825Ter	Nonsense	PI3K	Pathogenic
12	H	43	c.6219_6229delCTGCCATATTC	p.Cys2074PhefsTer10	Frameshift	FAT	Pathogenic
13	H	2	c.67C>T	p.Arg23Ter	Nonsense	N-terminal-TAN	Pathogenic
14	H	42	c.6100C>T	p.Arg2034Ter	Nonsense	FAT	Pathogenic
15	CH	2	c.67C>T	p.Arg23Ter	Nonsense	N-terminal-TAN	Pathogenic
		Intron 20–21	c.3077+1G>T	Splice site variant	Donor splicing variant	HEAT	Likely pathogenic
16	CH	37	c.5631_5635delCTCGCinsA	p.Phe1877LeufsTer39	Frameshift	HEAT	Pathogenic
		49	c.7307G>A	p.Arg2436Lys	Missense variant (affecting donor splice site)	FAT	VUS
17	H	52	c.7788G>C	p.Glu2596Asp	Missense variant (affecting donor splice site)	FAT-PI3K	VUS
18	CH	37	c.5631_5635delCTCGCinsA	p.Phe1877LeufsTer39	Frameshift	HEAT	Pathogenic
		49	c.7307G>A	p.Arg2436Lys	Missense variant (affecting donor splice site)	FAT	VUS
26	H	37	c.5631_5635delCTCGCinsA	p.Phe1877LeufsTer39	Frameshift	HEAT	Pathogenic

^#^Siblings; *CH* compound heterozygous, *H* homozygous, *PI3K* phosphatidylinositol 3-kinase, *FAT* FAT domain, *TAN* telomere-length maintenance and DNA damage repair domain, *VUS* variant of uncertain significance. *As per ACMG guidelines.

**Figure 6:**
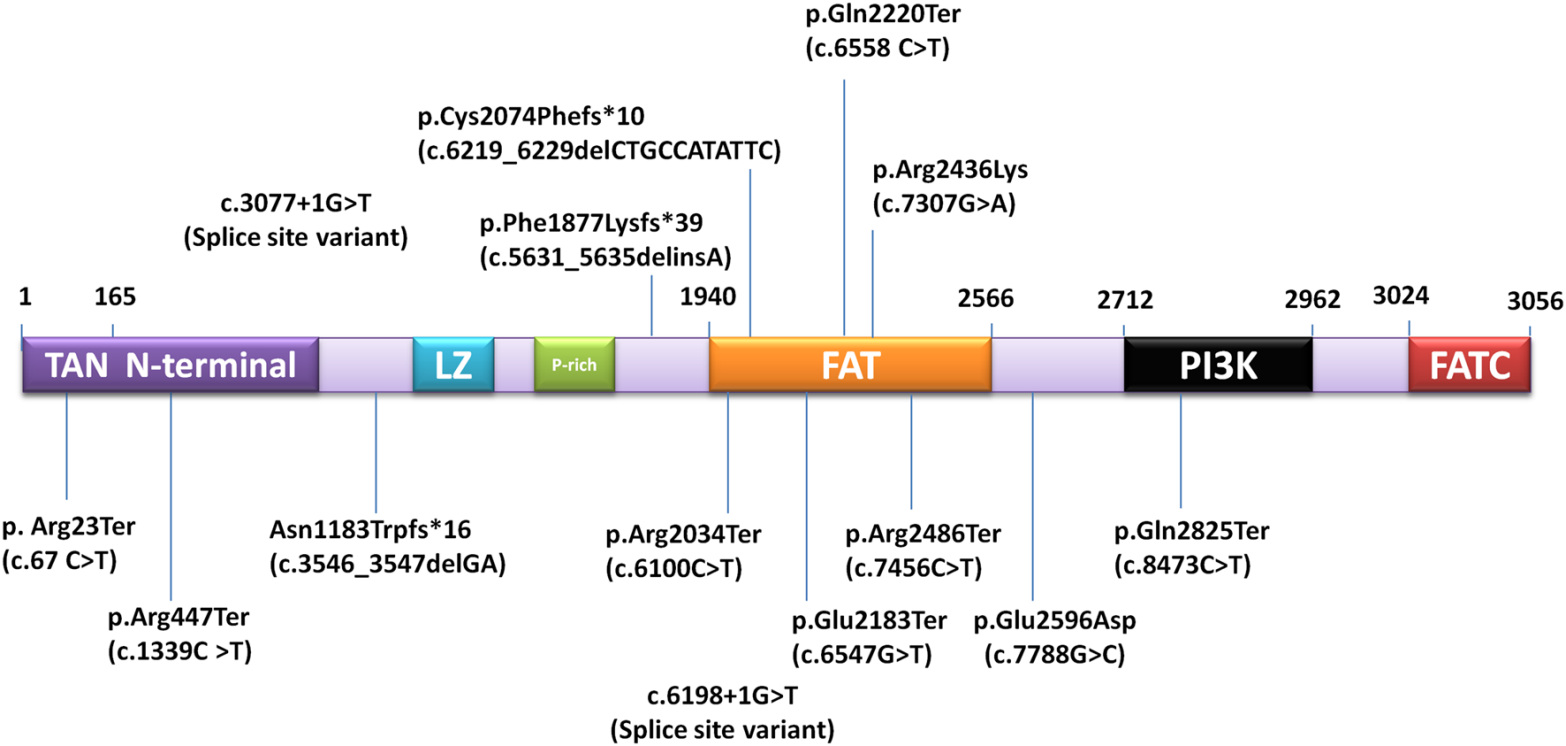
Domain wise depiction of *ATM* gene variants. *LZ* leucine zipper motif; phosphatidylinositol 3-kinase (PI3K), *FAT* FRAP-ATM-TRRAP, *FATC* FAT carboxy-terminal.

Seven of the 14 variants were found to be affecting the FAT domain, one affecting PI3K domain, and six variants were affecting the N-terminal domains. Position of one variant was observed to be between the FAT and PI3K domain. The most frequent variant c.67C>T affects the N-terminal Tel1/ATM N-Terminal Motif (TAN) of ATM protein. Figure 6 depicts the domain-wise location of *ATM* gene variants. Out of 8 A-T cases that harbored variants affecting FAT domain, 6 had homozygous variation and 2 had compound heterozygous biallelic variations. In the remaining 2 cases with bi-allelic variant (compound heterozygous state) at-least one allele affected the FAT domain. The details have been provided in Table 3.

## Discussion

Ataxia telangiectasia has remained an orphan disease in India. Due to variability in clinical manifestations, differential diagnosis becomes difficult and definite diagnosis is delayed. Recently, Mahadevappa et al. reported clinical features of 100 A-T cases and reported early-onset cerebellar ataxia with mucocutaneous telangiectasia as cardinal manifestations along with recurrent sinopulmonary infections, oculomotor abnormalities, and extrapyramidal involvement as common manifestations^10^. Unusual manifestations have previously been reported in the cases with A-T. Hyper IgM phenotype (elevated IgM with decreased IgG and IgA along with switch defect of B lymphocytes) has been observed in approximately 10% cases with A-T^11^. HIGM is also associated with severe immunological manifestations including autoimmunity, lymphoproliferation and poor outcomes^12^. Noordzij et al. studied the A-T cases with HIGM and proposed addition of DNA repair disorders as a possible cause of HIGM^11^. Various reports indicate that often A-T patients erroneously diagnosed as HIGM^11,13–16^. However, marked reduction of absolute T cell numbers along with elevated IgM and AFP could differentiate HIGM^11^. Minto et al. have also reported presence of cutaneous granulomas along with HIGM^17^. The presence of retinitis pigmentosa in the A-T cases has rarely been reported. Lymphoproliferations have previously been reported with A-T, however, its association with EBVs are rarely reported. Reyes et al. reported EBV associated laryngeal leiomyosarcoma and jejunal cellular leiomyoma in patient with A-T^18^. Majority of these cases also manifest immunodeficiency and may be related to immunosuppressive consequences. It would be critical to detect EBVs in A-T for better clinical management. Moreover, ATM protein could be implicated in the life cycle of EBV by using damage repair functions of ATM to facilitate replication^19^.

We herein report the spectrum of genetic variants in a North Indian A-T cohort. The majority of AT cases in our cohort harboured non-sense variants (56%) and small frameshift deletions or insertions (22%). The combined outcome from predominant (78%) stop-gain and frameshift variants in A-T results in the severe outcomes. Null mutations contribute to the morbidity and mortality of the A-T disease. This finding is consistent with previous findings that predominantly truncating variants contribute to loss of ATM protein in A-T^20–24^. Sandoval et al. reported nonsense and small frameshift deletions or insertions in more than half of the A-T cases investigated in their cohort^20^. The C → T transition was found to be the most common alteration in our cohort. The C → T transition is associated with methylation of cytosine residues in DNA^25,26^. These epigenetics mediated alterations are associated with CGA → TGA transitions leading to creation of stop codon^27,28^. The CGA (Arg) → TGA transitions are considered to be the most frequent nonsense mutations in the genome followed by CAG(Gln) → TAG transitions^29^. Therefore, GC rich regions have preponderance for hypermutability as described previously in model organisms^30^ and various other diseases^31,32^. CpG-rich loci have been associated with germline single nucleotide variations^33–35^.

Micol et al. reported that malignancies and respiratory tract infections were the major cause for mortality in the null and hypomorphic variants, respectively^36^. The c.67C>T variant was present in three unrelated patients among which two had homozygous and one had heterozygous variants (Five allelic variants). The germline variant at this position has also been associated with mantle cell lymphoma^37^, gastric cancer^38^ and A-T^39^ (ClinVar Accession: VCV000232248.14; Invitae: SCV000748724.5). The c.8473C>T variant was also found in two unrelated A-T cases. All other variants were private variants. Six variants are also being reported as a result of collaborative centralized rapid genetic diagnosis for severe combined immunodeficiency (SCID) in Japan (Manuscript in submission).

Initial studies, after decoding the sequence of *ATM* gene, identified many pathological variants^40^. Population-specific *ATM* variant spectrum has also been reported. Byrd et al. described six mutations affecting the N terminal part of ATM protein in the Irish population^41^. One of these variants was associated with a haplotype and was found to be common in four unrelated families. Similarly, Baumer et al. reported four novel variations in the cases with four different origins. One of the variants was found in the Turkish case that harbored homoallelic 174-bp deletion in the *ATM* gene due to consanguineous marriage^42^. Previous studies have also reported that the majority of variations in the *ATM* genes are heteroallelic^20,22,23^. However, similar to our cohort where 68.75% cases had homozygous variants, other recent studies have also reported homozygous variants in the patients with A-T^43,44^. Homoallelic variants in *ATM* gene develop in a population reflects founder effect. Kuznetsova et al. detected two novel variations in a Caucasian family, a mononucleotide deletion and a mononucleotide insertion, in children as well as prenatal testing in these families^45^.

Though population-based variability in the type of spectrum has been observed, the presence of nonsense and/or premature truncation has been a consistent finding. A recent study conducted in the Russian population reported founder mutations in the patients of Slavic origin with three recurrent mutations (c.5932G>T, c.450_453delTTCT, and c.1564_1565delGA) constituting 57% of total identified alleles^46^. The c.5932G>T (p.Glu1978*) allele was found to be the most frequent in the Russian population. Unlike the Russian population, missense (c.8147T>C) and splice site (c.3576G>A) variants were prominently identified to cause A-T in Dutch, Italian, German, and French cohort^47^. Moreover, the phenotype for these variants manifested as clinically milder A-T and reported longer survival and lesser susceptibility to having cancer, respiratory disease, and immunodeficiency in comparison to retrospectively identified Dutch classical AT cases. There were four variants (two splice site and two missense) in our cohort affecting the splicing event (c.6198+1G>T; c.3077+1G>T; c.7307G>A; c.7788G>C). The c.7307G>A; c.7788G>C alterations occur in the last base pair of coding exon 49 and exon 52, respectively. These alterations are predicted to affect the mRNA splicing event (Supplementary Table 2). Considering the highly conserved region amongst available vertebrate species the disruption of splicing event cause damage to the protein synthesis. In-silico splice site analysis predicts that these variants result in the splice donor site loss; Supplementary Table 2. However, functional studies are required to understand the pathogenicity of splicing variants^48^. Another variant (c.5631_5635delCTCGCinsA) has previously been reported in Iranian A–T patients^49^. Complex rearrangements sometimes could not be detected by the softwares and may cause challenge in identification of variants. However, extensive evaluation of BAM files through software like Integrative genomics viewer (IGV) and Detection of Exon Copy Number (DECoN) may help in detection of complex rearrangements. Large deletions/duplications can also be detected by using multiplex ligation-dependant probe amplification (MLPA)^50,51^ as previously reported^52,53^.

Various studies have reported the spectrum of *ATM* gene variants; however, there is dearth of genetic data of Indian A-T cases. Demuth et al. found the spectrum of known as well as novel variants in 19 A-T families of various ethnic backgrounds^54^. Recent case series from Sri Lanka reported six novel variants in four patients. Patients with truncating variants in this study had severe disease with an early onset^44^. A large report from Iran reported 9 novel variants out of 43 mutations in forty-three A-T cases^4^. Fievet et al. reported 49 *ATM* variants in 36 patients with typical and atypical A-T along with functional characterization of variants^55^. Various other studies have reported cases with atypical, delayed and/or mild clinic-biological presentations necessitating confirmation of pathogenicity of *ATM* variants^56–58^. Leuzzi et al. reported an atypical case of A-T who recovered from ataxia at the age of 12. She harboured compound heterozygous variation in *ATM* gene (Inframe deletion: p.Glu709_Lys750del42; Missense mutation: p.Asp2708Gln). The transcriptional profile of this case matched 90% to the classic A-T profile, however, 10% matched to healthy controls. Amongst the transcripts matched to healthy controls, two critical genes were *SCRB1* and *THOC3*. These genes are involved in wound repair, and genome stability, respectively. Another gene *THOC3* is known to be involved in transcriptional elongations, nuclear RNA export, and genome stability^59^. The matched transcript profile might be associated with the atypical manifestation of A-T phenotype. Lee et al. reported an atypical A-T harbouring compound heterozygous variant in A-T manifested with normal serum AFP, mild atrophy, delayed development and oculomotor apraxia^60^. These reports indicate that hypomorphic mutations are indeed associated with attenuated A-T phenotype^8,61,62^. McGrath-Morrow et al. associated epigenetic alterations with phenotypic variability in the patients with A-T^5^. However, this study does not vindicate that the differences in the DNA methylation profile between typical and atypical A-T is independent of the genotype. Variability of the phenotype may be associated with preserved function of ATM in the cases harbouring hypomorphic variants rather than independently due to epigenetic alterations.

In the absence of DNA damage, FAT and PI3K domains interact to form a dimer and inhibit the activity of ATM protein. In response to DNA damage, autophosphorylation of serine residue at 1981 location results in the activation of ATM protein due to dissociation of dimer^63^. The majority of mutant alleles in our cohort were confined to the region affecting FAT domain (at C-terminal) and a few affecting the HEAT sequences (N-terminal). HEAT sequences facilitate protein–protein interactions. Our cohort may have a severe outcome due to the involvement of FAT/PI3K domains and the presence of homozygous alterations. Three cases in our cohort with c.6547G>T *ATM* gene variation affecting FAT domain (position Glu2183), had onset at nine months of age. Another case with onset at 13 months had biallelic variant one of which (c.6100C>T) affected FAT domain at position Arg2034. Truncating variants have a huge impact on the structure, quantity and function of the protein and may be attributed to the severity in A-T. Four among five cases of our cohort who reported recurrent fever, loose stool, and infections harboured variants in the distal regions of the *ATM* gene and affected FAT or PI3K domains (p.Arg2436Lys; p.Glu2596Asp; p.Arg2486Ter; p.Gln2825Ter). However, the genetic analysis could not be established in one case.

ATM protein acts as a master regulator of double-stranded break response. The impairment in the response to DSBs leads to altered V(D)J recombination process which further results in cellular anomalies due to reduced end tethering; altered cell cycle checkpoints; programmed cell death^64^. In few AT cases, TREC assay indicates anomalies in thymic output and number of naive T cells. A few studies have reported incidental AT diagnosis on the new born screening for SCID. Mallott et al. reported reduced number of TRECs in 54% of AT cases^65^. Moreover, alterations in sjKREC in one case in our cohort indicate mutation centric anomalies in B cell neogenesis. The suboptimal interactions between T and B cells may mediate the course of immune dysfunction^66^. Giovannetti et al. have described presence of terminally differentiated T cells and skewed T cell receptor repertoire due to *ATM* gene variants^67^. We also reported enhanced T cell immunosenescence especially in the T cytotoxic cells.

We could not study the role of signalling cascade of MRE11/RAD50/NBN (MRN) complex in our A-T cohort. The molecular expression and interaction of ATM protein with MRN complex influence the clinical severity. The MRN complex recruits ATM protein upon sensing the DSBs and facilitates repair. Moreover, MRN complex facilitates telomere stability^68–70^ and may involve in the immunosenescence observed in the A-T cases. Epigenetic modifying factors may also govern the A-T phenotype and provide resilience to some cases. Murine RAD50^s^ model has shown alleviation of classic A-T manifestations including senescence, radiosensitivity, and malignancy^71^.

Since, this was a retrospective analysis, some of the patients in the cohort are now non-ambulatory and not coming for follow-up. We could not firmly establish in the case of compound heterozygous variants in patients whether they were in cis or trans. We could only predict the functional impact of splice site variants using in-silico prediction tools but could not perform transcript analysis in four cases with splice site variants. Moreover, large genomic rearrangements including deletions/duplications could not be performed by alternative methods such as MLPA. Oncologic risk management could not be established in consanguineous parents.

We report the data of 26 cases clinically correlated with Ataxia-Telangiectasia phenotype according to the hallmark clinical and biochemical markers along with genetic analysis in sixteen unrelated cases. To the best of our knowledge, this is the first study reporting genetic spectrum and T-cell receptor excision circles data of Indian cases with Ataxia Telangiectasia. Current retrospective analysis limits us to deduce the temporal change in severity due to limited data. The available data reveal higher null and truncating variants, predominantly reduced IgA, as well as hyper IgM-like phenotype in two cases, altered TRECs and sjKRECs, indicating an underlying immune defect. However, a larger cohort with classical and atypical A-T phenotypes would confirm these findings or give a clearer picture of the spectrum of *ATM* mutations in North India.

## Supplementary Information

Supplementary Information.

## Data Availability

Data will be made available on request.

## References

[1] Rothblum-Oviatt C (2016). Ataxia telangiectasia: A review. Orphanet. J. Rare Dis..

[2] Lee SJ (2005). Identification and functional analysis of cystathionine beta-synthase gene mutations in patients with homocystinuria. J. Hum. Genet..

[3] Gatti RA (1988). Localization of an ataxia-telangiectasia gene to chromosome 11q22-23. Nature.

[4] Amirifar P (2021). The spectrum of ATM gene mutations in Iranian patients with ataxia-telangiectasia. Pediatr. Allergy Immunol..

[5] McGrath-Morrow SA (2020). DNA methylation and gene expression signatures are associated with ataxia-telangiectasia phenotype. Sci. Rep..

[6] Malaviya AN, Sachdeva KK, Singh N (1973). Ataxia telangiectasia: Immunological abnormalities in probands and first degree relatives in 5 families. J. Assoc. Physicians India.

[7] Staples ER (2008). Immunodeficiency in ataxia telangiectasia is correlated strongly with the presence of two null mutations in the ataxia telangiectasia mutated gene. Clin. Exp. Immunol..

[8] Verhagen MM (2012). Presence of ATM protein and residual kinase activity correlates with the phenotype in ataxia-telangiectasia: A genotype-phenotype study. Hum. Mutat..

[9] Morinishi Y (2009). Identification of severe combined immunodeficiency by T-cell receptor excision circles quantification using neonatal guthrie cards. J. Pediatr..

[10] Mahadevappa M, Kamble N, Santhosh KDV, Yadav R, Netravathi M, Pal PK (2020). A clinical profile of 100 patients with ataxia telangiectasia seen at a tertiary care center. Ann. Mov. Disord..

[11] Noordzij JG (2009). Ataxia-telangiectasia patients presenting with hyper-IgM syndrome. Arch. Dis. Child.

[12] Ghiasy S (2017). The clinical significance of complete class switching defect in Ataxia telangiectasia patients. Expert Rev. Clin. Immunol..

[13] Doshi A (2016). Ataxia telangiectasia presenting as hyper IgM syndrome without neurologic signs. Ann. Allergy Asthma Immunol..

[14] Rawat A (2016). Ataxia telangiectasia masquerading as hyper IgM syndrome. Indian J. Pediatr..

[15] Aghamohammadi A (2010). Ataxia-telangiectasia in a patient presenting with hyper-immunoglobulin M syndrome. J. Investig. Allergol. Clin. Immunol..

[16] Pietrucha BM (2010). Ataxia-telangiectasia with hyper-IgM and Wilms tumor: Fatal reaction to irradiation. J. Pediatr. Hematol. Oncol..

[17] Minto H (2019). A novel ATM mutation associated with elevated atypical lymphocyte populations, hyper-IgM, and cutaneous granulomas. Clin. Immunol..

[18] Reyes C, Abuzaitoun O, De Jong A, Hanson C, Langston C (2002). Epstein–Barr virus-associated smooth muscle tumors in ataxia-telangiectasia: A case report and review. Hum. Pathol..

[19] Tatfi M, Hermine O, Suarez F (2018). Epstein–Barr virus (EBV)-related lymphoproliferative disorders in ataxia telangiectasia: Does ATM regulate EBV life cycle?. Front. Immunol..

[20] Sandoval N (1999). Characterization of ATM gene mutations in 66 ataxia telangiectasia families. Hum. Mol. Genet..

[21] Gilad S (1996). Predominance of null mutations in ataxia-telangiectasia. Hum. Mol. Genet..

[22] Huang Y (2013). Twelve novel Atm mutations identified in Chinese ataxia telangiectasia patients. Neuromol. Med..

[23] Concannon P, Gatti RA (1997). Diversity of ATM gene mutations detected in patients with ataxia-telangiectasia. Hum. Mutat..

[24] Wright J (1996). A high frequency of distinct ATM gene mutations in ataxia-telangiectasia. Am. J. Hum. Genet..

[25] Ehrlich M, Norris KF, Wang RY, Kuo KC, Gehrke CW (1986). DNA cytosine methylation and heat-induced deamination. Biosci. Rep..

[26] Cooper DN, Krawczak M (1989). Cytosine methylation and the fate of CpG dinucleotides in vertebrate genomes. Hum. Genet..

[27] Romanov GA, Sukhoverov VS (2017). Arginine CGA codons as a source of nonsense mutations: A possible role in multivariant gene expression, control of mRNA quality, and aging. Mol. Genet. Genomics.

[28] Cooper DN, Mort M, Stenson PD, Ball EV, Chuzhanova NA (2010). Methylation-mediated deamination of 5-methylcytosine appears to give rise to mutations causing human inherited disease in CpNpG trinucleotides, as well as in CpG dinucleotides. Hum. Genomics.

[29] Mort M, Ivanov D, Cooper DN, Chuzhanova NA (2008). A meta-analysis of nonsense mutations causing human genetic disease. Hum. Mutat..

[30] Kiktev DA, Sheng Z, Lobachev KS, Petes TD (2018). GC content elevates mutation and recombination rates in the yeast *Saccharomyces**cerevisiae*. Proc. Natl. Acad. Sci. U.S.A..

[31] Schmegner C, Hoegel J, Vogel W, Assum G (2007). The rate, not the spectrum, of base pair substitutions changes at a GC-content transition in the human NF1 gene region: Implications for the evolution of the mammalian genome structure. Genetics.

[32] Kaur R, Attri SV, Saini AG, Sankhyan N (2021). A high frequency and geographical distribution of MMACHC R132* mutation in children with cobalamin C defect. Amino Acids.

[33] Nesta AV, Tafur D, Beck CR (2021). Hotspots of human mutation. Trends Genet.

[34] Shendure J, Akey JM (2015). The origins, determinants, and consequences of human mutations. Science.

[35] Coulondre C, Miller JH, Farabaugh PJ, Gilbert W (1978). Molecular basis of base substitution hotspots in *Escherichia**coli*. Nature.

[36] Micol R (2011). Morbidity and mortality from ataxia-telangiectasia are associated with ATM genotype. J. Allergy Clin. Immunol..

[37] Camacho E (2002). ATM gene inactivation in mantle cell lymphoma mainly occurs by truncating mutations and missense mutations involving the phosphatidylinositol-3 kinase domain and is associated with increasing numbers of chromosomal imbalances. Blood.

[38] Huang DS (2015). Prevalence of deleterious ATM germline mutations in gastric cancer patients. Oncotarget.

[39] Amirifar P (2021). The spectrum of ATM gene mutations in Iranian patients with ataxia-telangiectasia. Pediatr. Allergy Immunol..

[40] Vorechovsky I (1996). Exon-scanning mutation analysis of the ATM gene in patients with ataxia-telangiectasia. Eur. J. Hum. Genet..

[41] Byrd PJ (1996). Mutations revealed by sequencing the 5′ half of the gene for ataxia telangiectasia. Hum. Mol. Genet..

[42] Baumer A, Bernthaler U, Wolz W, Hoehn H, Schindler D (1996). New mutations in the ataxia telangiectasia gene. Hum. Genet..

[43] Chen W (2019). Novel homozygous ataxiatelangiectasia (AT) mutated gene mutation identified in a Chinese pedigree with AT. Mol. Med. Rep..

[44] Hettiarachchi D (2020). Six Novel ATM gene variants in Sri Lankan patients with ataxia telangiectasia. Case Rep. Genet..

[45] Kuznetsova MV (2017). Two novel mutations associated with ataxia-telangiectasia identified using an ion AmpliSeq inherited disease panel. Front. Neurol..

[46] Suspitsin E (2020). ATM mutation spectrum in Russian children with ataxia-telangiectasia. Eur. J. Med. Genet..

[47] van Os NJH (2019). Genotype-phenotype correlations in ataxia telangiectasia patients with ATM c.3576G>A and c.8147T>C mutations. J. Med. Genet..

[48] Gelman H (2019). Recommendations for the collection and use of multiplexed functional data for clinical variant interpretation. Genome Med..

[49] Babaei M, Mitui M, Olson ER, Gatti RA (2005). ATM haplotypes and associated mutations in Iranian patients with ataxia-telangiectasia: Recurring homozygosity without a founder haplotype. Hum. Genet..

[50] Tyagi R (2019). Repurposing pathogenic variants of DMD gene and its isoforms for DMD exon skipping intervention. Curr. Genomics.

[51] Tyagi R, Aggarwal P, Mohanty M, Dutt V, Anand A (2020). Computational cognitive modeling and validation of Dp140 induced alteration of working memory in Duchenne Muscular Dystrophy. Sci. Rep..

[52] Cavalieri S (2008). Large genomic mutations within the ATM gene detected by MLPA, including a duplication of 41 kb from exon 4 to 20. Ann. Hum. Genet..

[53] Cavalieri S (2006). ATM mutations in Italian families with ataxia telangiectasia include two distinct large genomic deletions. Hum. Mutat..

[54] Demuth I (2011). New mutations in the ATM gene and clinical data of 25 AT patients. Neurogenetics.

[55] Fievet A (2019). Functional classification of ATM variants in ataxia-telangiectasia patients. Hum. Mutat..

[56] Meissner WG (2013). Isolated generalized dystonia in biallelic missense mutations of the ATM gene. Mov. Disord..

[57] Keimling M (2011). Functional characterization connects individual patient mutations in ataxia telangiectasia mutated (ATM) with dysfunction of specific DNA double-strand break-repair signaling pathways. FASEB J..

[58] Dork T, Bendix-Waltes R, Wegner RD, Stumm M (2004). Slow progression of ataxia-telangiectasia with double missense and in frame splice mutations. Am. J. Med. Genet. A.

[59] Leuzzi V (2018). Ataxia-telangiectasia: A new remitting form with a peculiar transcriptome signature. Neurol. Genet..

[60] Lee HY (2021). Compound heterozygous variants including a novel copy number variation in a child with atypical ataxia-telangiectasia: A case report. BMC Med. Genomics.

[61] Mitui M (2009). Functional and computational assessment of missense variants in the ataxia-telangiectasia mutated (ATM) gene: Mutations with increased cancer risk. Hum. Mutat..

[62] Saviozzi S (2002). A late onset variant of ataxia-telangiectasia with a compound heterozygous genotype, A8030G/7481insA. J. Med. Genet..

[63] Bakkenist CJ, Kastan MB (2003). DNA damage activates ATM through intermolecular autophosphorylation and dimer dissociation. Nature.

[64] Weitering TJ, Takada S, Weemaes CMR, van Schouwenburg PA, van der Burg M (2021). ATM: Translating the DNA damage response to adaptive immunity. Trends Immunol..

[65] Mallott J (2013). Newborn screening for SCID identifies patients with ataxia telangiectasia. J. Clin. Immunol..

[66] Driessen GJ (2013). Antibody deficiency in patients with ataxia telangiectasia is caused by disturbed B- and T-cell homeostasis and reduced immune repertoire diversity. J. Allergy Clin. Immunol..

[67] Giovannetti A (2002). Skewed T-cell receptor repertoire, decreased thymic output, and predominance of terminally differentiated T cells in ataxia telangiectasia. Blood.

[68] Lavin MF, Kozlov S, Gatei M, Kijas AW (2015). ATM-dependent phosphorylation of all three members of the MRN complex: From sensor to adaptor. Biomolecules.

[69] Stingele J, Bellelli R, Boulton SJ (2017). Mechanisms of DNA-protein crosslink repair. Nat. Rev. Mol. Cell Biol..

[70] Williams GJ, Lees-Miller SP, Tainer JA (2010). Mre11-Rad50-Nbs1 conformations and the control of sensing, signaling, and effector responses at DNA double-strand breaks. DNA Repair (Amst).

[71] Morales M (2005). The Rad50S allele promotes ATM-dependent DNA damage responses and suppresses ATM deficiency: Implications for the Mre11 complex as a DNA damage sensor. Genes Dev..

