# Supplementary material for: Unusual clinical manifestations and predominant stopgain ATM gene variants in a single centre cohort of ataxia telangiectasia from North India
Source: Sci Rep. 2022 Mar 8;12:4036. doi: 10.1038/s41598-022-08019-0 (PMC8904522; doi:10.1038/s41598-022-08019-0)
Supplement: Supplementary file 1 — Supplementary Information. [file 41598_2022_8019_MOESM1_ESM.docx]

Title: Unusual clinical manifestations and predominant stopgain *ATM* gene variants in a single centre cohort of ataxia telangiectasia from North India

**Authors and affiliations:**

Amit Rawat^1*,^ ^#^, Rahul Tyagi^1, #^, Himanshi Chaudhary^1^, Vignesh Pandiarajan^1^, Ankur Kumar Jindal^1^, Deepti Suri^1^, Anju Gupta^1^, Madhubala Sharma^1^, Kanika Arora^1^, Amanjit Bal^2^, Priyanka Madaan^3^, Lokesh Saini^3^, Jitendra Kumar Sahu^3^, Yumi Ogura^4^, Tamaki Kato^4^, Kohsuke Imai^4,5^, Shigeaki Nonoyama^4^, Surjit Singh^1^

^1^Allergy and Immunology Unit, Department of Pediatrics, Postgraduate Institute of Medical Education and Research, Chandigarh, India. ^2^Department of Histopathology, Postgraduate Institute of Medical Education and Research, Chandigarh, India. ^3^Pediatric Neurology Unit, Department of Pediatrics, Post Graduate Institute of Medical Education and Research, Chandigarh, India. ^4^National Defense Medical College (Japan), Japan. ^5^Tokyo Medical and Dental University, Japan

**Supplementary Table 1: Levels of TREC, SJKREC and CJKREC in the patients with A-T.**

| S. No | Case | TREC | SJKREC | CJKREC | RNAseP |
| --- | --- | --- | --- | --- | --- |
| 1 | P4 | 97.73 | 17509.93 | 124249.21 | 2051308.15 |
| 2 | P5 | 47.36 | 40682.75 | 188013.35 | 1598984.77 |
| 3 | P6 | 75.95 | 91706.14 | 213645.07 | 1668354.43 |
| 4 | P7 | 0 | 8350 | 37500 | 1730000 |
| 5 | P11 | 185 | 3820 | 17000 | 1650000 |
| 6 | P12 | 12.81 | 18785.31 | 174670.43 | 3215630.89 |
| 7 | P16 | 0 | 0 | 2500 | 1640000 |
| 8 | P25 | 0 | 27000 | 18000 | 2650000 |

**Supplementary Table 2: Predications of functional impact of variants on splicing mechanism in the patients with A-T**

| S. No | Variants* | SpliceAI - Donor Loss | Maxentscan  (alt) | dbscSNV (ADA) | CADD-  Phred | MPA-Score; Impact | ClinVar  (Accession ID) | ACMG |
| --- | --- | --- | --- | --- | --- | --- | --- | --- |
| 1 | c.6198+1G>T  (Intronic) | 0.99 | -0.710 | 0.9999 | 34.00 | 10; high | Absent | Likely pathogenic (PVS1+PM2) |
| 2 | c.3077+1G>T  (Intronic) | 0.99 | -1.474 | 0.9999 | 35.00 | 10; high | Probably pathogenic (SCV001377084.2) | Likely pathogenic (PVS1+PM2) |
| 3 | c.7307G>A (p.Arg2436Lys) | 0.94 | 4.446 | 0.9999 | 33.00 | 10; high | Conflicting interpretations of pathogenicity (VCV000187003.7) | VUS (PM2+PM1+PP3) |
| 4 | c.7788G>C  (p.Glu2596Asp) | 0.81 | 2.138 | 0.9999 | 32.00 | 10; high | Uncertain significance​ (VCV000558043.1) | VUS (PM2+PM5+PP3) |

SpliceAI: Donor Loss: thresholds > 0.2|0.5|0.8 for impact; MaxEntScan^1^: high (alt < 6.2), moderate (6.2 ≤ alt ≤ 8.5) or low (alt > 8.5); dbscSNV (ADA) 0 (low impact) and 1 (High impact); MoBiDiC Prioritization Algorithm (MPA) Raw score [0; 10], 10: high impact. *Transcript analysis could not be performed for these variants. ACMG: American College of Medical Genetics and Genomics; VUS: Variant of uncertain significance.

Supplementary Figure 1

Supplementary Figure 1: Integrative Genome Viewer (IGV) based analysis revealing the variant c.5631_5635delCTCGCinsA in A) P18 (heterozygous) and B) P26 (homozygous).

**Supplementary Figure-2**


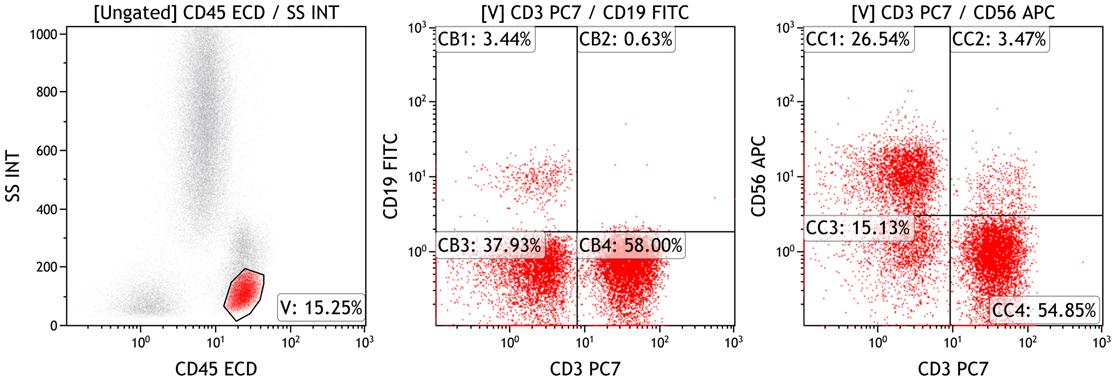


**Supplementary Figure 2:** Lymphocyte subset of the patient P26 revealing reduced proportion of CD19+ B cells (3.44%, red arrow at CB1). The lymphocytes were gated on SSc vs CD45 (Pan-leukocyte marker). CD3+ T cells (CC4), CD19+B cells (CB1) and CD56+NK cells (CC1) were gated on CD45+ lymphocytes (V). Normal age appropriate reference range for CD19+ B cells were (13-27%).

**Supplementary Figure 3:**


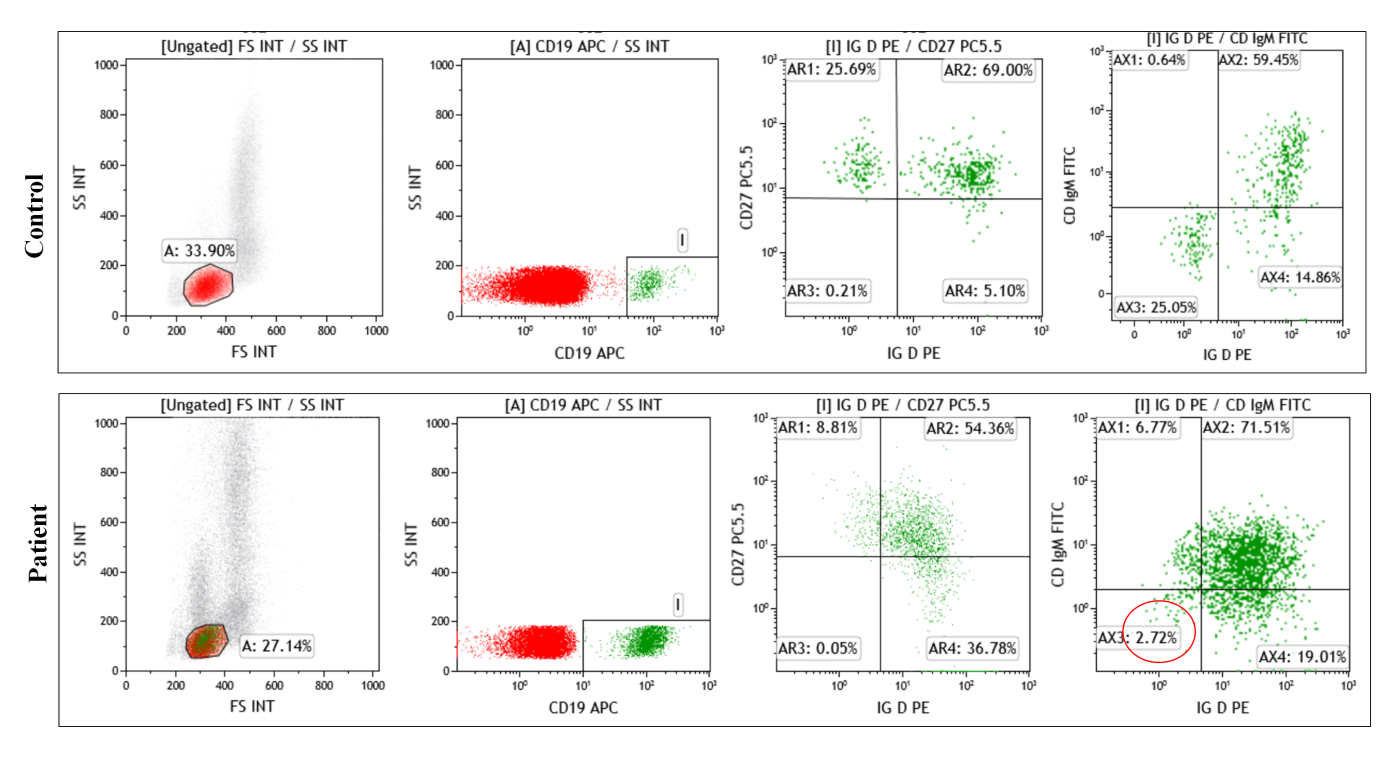


**Supplementary Figure 3:** B cell immunophenotyping in the patient (P15) with Ataxia Telangiectasia revealed reduced proportion (2.72%) of CD19+IgM-IgD- switched B lymphocytes (highlighted in red circle, in comparison to control (25.05%) at similar location (AX3). Lymphocytes were gated on FSc vs SSc. B cells (CD19+) were gated on lymphocytes (A). CD19+CD27-IgD+ Naïve B cells (AR4), CD19+CD27+IGD+ Unswitched memory (AR2) and CD19+CD27+IGD- switched memory B cells (AR1) were gated on CD19+B cells (I). CD19+IgM-IgD- switched B lymphocytes were also gated on CD19+ B cells (I). The age appropriate normal range for switched B cells were (6.7-31.1% of CD19+cells)^2^.

Reference:

1 Shamsani, J. *et al.* A plugin for the Ensembl Variant Effect Predictor that uses MaxEntScan to predict variant spliceogenicity. *Bioinformatics* **35**, 2315-2317, doi:10.1093/bioinformatics/bty960 (2019).

2 Besci, O. *et al.* Reference values for T and B lymphocyte subpopulations in Turkish children and adults. *Turk J Med Sci* **51**, 1814-1824, doi:10.3906/sag-2010-176 (2021).
